# Surgical manipulation compromises leukocyte mobilization responses and inflammation after experimental cerebral ischemia in mice

**DOI:** 10.3389/fnins.2013.00271

**Published:** 2014-01-17

**Authors:** Adam Denes, Jesus M. Pradillo, Caroline Drake, Hannah Buggey, Nancy J. Rothwell, Stuart M. Allan

**Affiliations:** ^1^Faculty of Life Sciences, University of ManchesterManchester, UK; ^2^Laboratory of Molecular Neuroendocrinology, Institute of Experimental MedicineBudapest, Hungary

**Keywords:** experimental stroke, bone marrow, granulocyte, CXCR2, anaesthesia

## Abstract

Acute brain injury results in peripheral inflammatory changes, although the impact of these processes on neuronal death and neuroinflammation is currently unclear. To facilitate the translation of experimental studies to clinical benefit, it is vital to characterize the mechanisms by which acute brain injury induces peripheral inflammatory changes, and how these are affected by surgical manipulation in experimental models. Here we show that in mice, even mild surgical manipulation of extracranial tissues induced marked granulocyte mobilization (300%) and systemic induction of cytokines. However, intracranial changes induced by craniotomy, or subsequent induction of focal cerebral ischemia were required to induce egress of CXCR2-positive granulocytes from the bone marrow. CXCR2 blockade resulted in reduced mobilization of granulocytes from the bone marrow, caused an unexpected increase in circulating granulocytes, but failed to affect brain injury induced by cerebral ischemia. We also demonstrate that isoflurane anaesthesia interferes with circulating leukocyte responses, which could contribute to the reported vascular and neuroprotective effects of isoflurane. In addition, no immunosuppression develops in the bone marrow after experimental stroke. Thus, experimental models of cerebral ischemia are compromised by surgery and anaesthesia in proportion to the severity of surgical intervention and overall tissue injury. Understanding the inherent confounding effects of surgical manipulation and development of new models of cerebral ischemia with minimal surgical intervention could facilitate better understanding of interactions between inflammation and brain injury.

## Introduction

Brain injury due to cerebral ischemia, hemorrhage, or head trauma results in early activation of the immune system, followed later by immunosuppression (Dirnagl et al., 2007; Denes et al., 2010b). Activated immune cells and inflammatory mediators contribute to neuroinflammation and overall outcome after experimental brain injury. Blockade of toll-like receptors, proinflammatory mediators such as interleukin-1, and immune cells such as T cells, neutrophils, or mast cells, is protective in experimental models of brain injury (Iadecola and Anrather, 2011; Smith et al., 2013). However, oversuppression of the immune system in response to brain injury gives rise to opportunistic infections leading to impaired recovery and death of patients and experimental animals (Dirnagl et al., 2007; Murray et al., 2013). Therefore, understanding interactions between peripheral inflammatory responses and brain injury could pave the way for novel therapeutic interventions.

Lack of translation in stroke has triggered intense discussions, leading to improved preclinical guidelines on experimental modeling (Fisher et al., 2009). Yet, compared to the large number of clinical studies on peripheral inflammatory changes in stroke, studies on systemic inflammatory mechanisms in experimental animals remain relatively sparse.

We and others have shown that experimental stroke results in the activation of inflammatory responses in various immune organs such as the spleen, blood, or bone marrow (Offner et al., 2006a,b; Chapman et al., 2009; Denes et al., 2011). Using a filament model of transient, focal cerebral ischemia we also showed that anaesthesia and surgical intervention compromise stroke-induced inflammatory responses in the periphery (Denes et al., 2011). However, a systematic analysis of stroke-induced peripheral inflammatory actions, including the assessment of confounding effects of surgical manipulations has not yet been performed in experimental stroke that involves craniotomy. Such investigations could distinguish model-specific and ischemia-induced effects of experimental stroke on immune responses and yield important data for translation to patients.

Thus, we examined the effects of anaesthesia, surgical manipulation, and experimental stroke induced by transient, distal middle cerebral artery occlusion with craniotomy on peripheral inflammatory responses. We show that both isoflurane anaesthesia and surgical intervention induce changes in peripheral immune cell populations in the absence of stroke, and are likely to contribute to systemic inflammatory responses induced by experimental brain injury. These effects should be accounted for in experimental stroke modeling.

## Materials and methods

### Mice

Male 8–12 week-old C57BL/6 mice (*n* = 58) were kept at 21 ± 1°C and 65% humidity with a 12 h light-dark cycle and had free access to food and water. All animal procedures were performed under appropriate project license authority and adhered to the UK Animals (Scientific Procedures) Act (1986) and were in accordance with STAIR and ARRIVE (Fisher et al., 2009; Kilkenny et al., 2010) guidelines.

### Middle cerebral artery occlusion (MCAo)

Distal, transient focal cerebral ischemia was induced as described earlier (Pradillo et al., 2009, 2012). Briefly, mice were anaesthetized with isoflurane and ischemia was induced by a transient ligature (60 min) of the left MCA trunk with a 10-0 suture (Prolene, Ethicon, Somerville, NJ, USA). Occlusion and reperfusion were confirmed visually under the surgical microscope. Core body temperature was maintained at 37.0 ± 0.5°C throughout the surgery by a heating blanket (Homeothermic Blanket Control Unit; Harvard Apparatus, Kent, UK) and monitored after recovery. After surgery, animals were returned to their cages and allowed free access to water and food. It was decided, *a priori*, to exclude from the study those animals that showed brain hemorrhage at any time of the surgery or with no reperfusion (2 mice). The survival rate was 100%.

### Surgical controls

We investigated the effect of surgical manipulation and anaesthesia in the absence of MCAo on cellular and cytokine responses. To achieve this, three separate experimental conditions were used; the first involved only anaesthesia with no surgical manipulation (termed as “isoflurane”), the second exposure of the skull bone but no craniotomy (termed as “sham no cran.”) and the third full craniotomy (termed as “sham”). Except for naive animals, all groups of mice were kept under isoflurane anaesthesia for the same time period (75 min for control experimental conditions and in the “MCAo” group).

### Blood sampling and tissue processing

**Figure 1:**
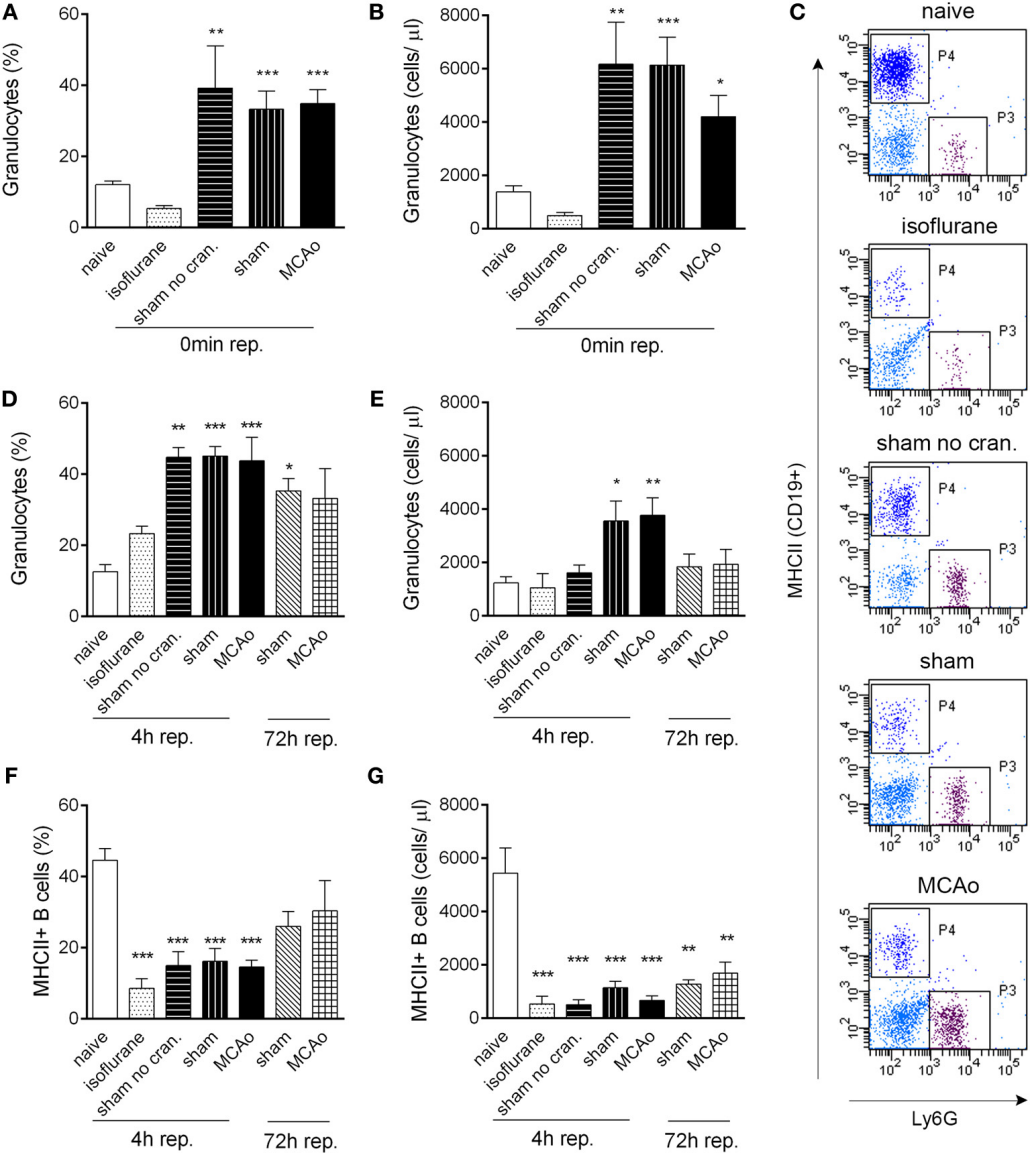
**Tissue injury, craniotomy, and anaesthesia result in rapid alteration of leukocyte responses**. Extracranial tissue injury results in increased proportion **(A)** and cell numbers **(B)** of granulocytes in the circulation, which is indistinguishable from changes induced by sham surgery or MCAo. Blood samples taken from the tail vein prior to surgery and 60 min after the onset of MCAo/control surgery (0 min reperfusion) are shown. **(C)** Representative dot plots showing terminal cardiac blood samples at 4 h reperfusion for graphs **(D–G)**. P3 gate: Ly6G+ granulocytes, P4 gate: MHCII+ B cells (dark blue color indicates CD19+ positive cells). Proportion **(D)** and total cell numbers **(E)** of granulocytes in cardiac blood samples at 4 and 72 h reperfusion. Proportion **(F)** and total cell numbers **(G)** of MHCII+ B cells (CD19+) in cardiac blood samples at 4 and 72 h reperfusion. Sham no cran.—sham surgery without craniotomy. *n* = 5, 4, 4, 5, 6–7, 3, 3 in naïve, isoflurane, sham no cran., sham, MCAo, sham 72 h, and MCAo 72 h, respectively. ^*^P < 0.05, ^**^P < 0.01, ^***^P < 0.001 vs. naive. Light blue color indicates negative (unstained) population.

Blood samples were taken from the tail vein and from the right cardiac ventricle, using 3.8% sodium citrate 1:10 as an anticoagulant. Repeated tail vein samples were collected at various time points, with equal sampling times across different treatment groups: prior to surgery (“naïve”), 60 min after the onset of ischemia or corresponding control surgery (“0 min reperfusion”), and before transcardial perfusion (at 4 or 72 h reperfusion, not shown). Terminal cardiac blood samples were collected immediately prior to transcardial perfusion from naive mice, and from mice that had undergone surgical interventions, at 4 or 72 h reperfusion. Blood sample data from different vascular beds were analyzed separately. To avoid potential confounding effects of repeated tail vein samples at later time points, cardiac blood samples are presented throughout the manuscript, with the exception of Figures 1A,B. To isolate the bone marrow and the spleen, mice were perfused transcardially with saline under isoflurane anaesthesia. Brains were subsequently perfused with 4% paraformaldehyde, post-fixed for 24 h, cryoprotected in 20% sucrose/PBS, and sectioned (20 μm diameter) on a sledge microtome. Organs were homogenized as described previously (Denes et al., 2010a).

### CXCR2 blockade

To prevent CXCR2-mediated release of granulocytes from the bone marrow, mice in the MCAo group were treated intraperitoneally with a selective CXCR2 antagonist (SB225002) or vehicle. SB225002 was dissolved in DMSO (stock), diluted in sterile saline 1:40 and injected as 2 mg/kg, 200 μ l/mouse, 20 min prior to induction of anaesthesia for MCAo surgery.

### *In vitro* stimulation of bone marrow cells

Bone marrow cells were isolated 72 h after 60 min MCAo or the surgery control and stimulated *in vitro* with 1 μg/ml bacterial lipopolysaccharide (LPS, *E. coli* O26:B6), at a density of 7.5 × 10^6^ cells/mL in RPMI medium, supplemented with 10% fetal calf serum and penicillin/streptomycin to investigate cytokine production. After 3 h incubation at 37°C, cells were pelleted with centrifugation at 400 × *g*, the supernatant was collected, and cells were lysed in lysis buffer.

### Cytometric bead array

Circulating levels of IL-6 and KC (CXCL-1), and levels of IL-6 in bone marrow cell culture supernatants and lysates were measured by using CBA Flex Sets (BD Biosciences, UK) according to the manufacturers protocol. The detection limit for each cytokine was 5–10 pg/ml.

### Flow cytometry

Spleen, bone marrow, and blood cells were isolated and stained with appropriate combinations of CD45-PerCP-Cy5.5, Ly6c-PerCP-Cy5.5, CD4-PE-Cy7, CD8-PE, CD3-APC, CD19-PE-Cy7, MHCII-APC (eBioscience, UK), and Ly6G-PE (1A8, BD Biosciences, UK) following Fc receptor blockade (eBioscience). Contaminating red blood cells in bone marrow and spleen samples were removed by ACK solution, and FACS lysing solution (BD Biosciences) was used to remove red blood cells from blood samples. Total blood cell counts were calculated by using 15 μm polystyrene microbeads (Polysciences, 18328-5). Cells were acquired on an LSRII flow cytometer, using FACS Diva software (BD Biosciences, UK). Except for the 72 h time point, flow cytometric data have been pooled from three independent experiments, in which all experimental groups have been represented by at least one animal. Due to red blood cell contamination and cell labeling artifacts *n* = 6 blood samples, *n* = 3 bone marrow samples and *n* = 4 spleen samples have been excluded from analysis across experiments, *pre-hoc*.

### Randomization, quantification, and statistical analysis

Animals were randomized for experiments and all quantitative analyses were performed in a blinded manner. Group sizes were determined based on our earlier *in vivo* experiments using this experimental model. Normality of data sets was assessed using GraphPad Prism (KS normality test). Statistical analysis was performed by One-Way or Two-Way ANOVA followed by Tukey's or Bonferroni's *post-hoc* multiple-comparison, using GraphPad Prism 5 software. In case of non-parametric data, significance was confirmed by non-parametric *t*-test (Mann–Whitney *U*-test) or Kruskal–Wallis test followed by Dunn's multiple comparisons test. All data are expressed as mean ± standard error of the mean (s.e.m). *P* < 0.05 was considered statistically significant.

## Results

### Surgical manipulation and anaesthesia alter leukocyte responses in the blood

Surgical manipulation of extracranial tissues was sufficient to induce rapid mobilization of granulocytes. In serial blood samples taken from the tail vein, the proportion of granulocytes (Ly6G+ CD11b+ SSC^high^ cells) increased from 12 to 40% within 70–80 min (“0 min reperfusion”) in response surgery without craniotomy (“sham no cran.,” Figure 1A), but not after isoflurane anaesthesia alone, representing a ~3-fold increase in total granulocyte numbers (Figure 1B). Granulocyte mobilization was not altered further by craniotomy or MCAo (Figures 1A,B). Granulocytosis was also evident 5–5.5 h after initiation of surgery in cardiac, terminal blood samples (termed as “4 h reperfusion,” Figures 1C,D), although total granulocyte numbers were lower compared to the earlier time point and were significantly elevated only in response to craniotomy or MCAo (Figure 1E). An increased proportion of granulocytes in the sham and MCAo groups was observed up to 72 h after surgery (Figure 1D).

**Figure 2:**
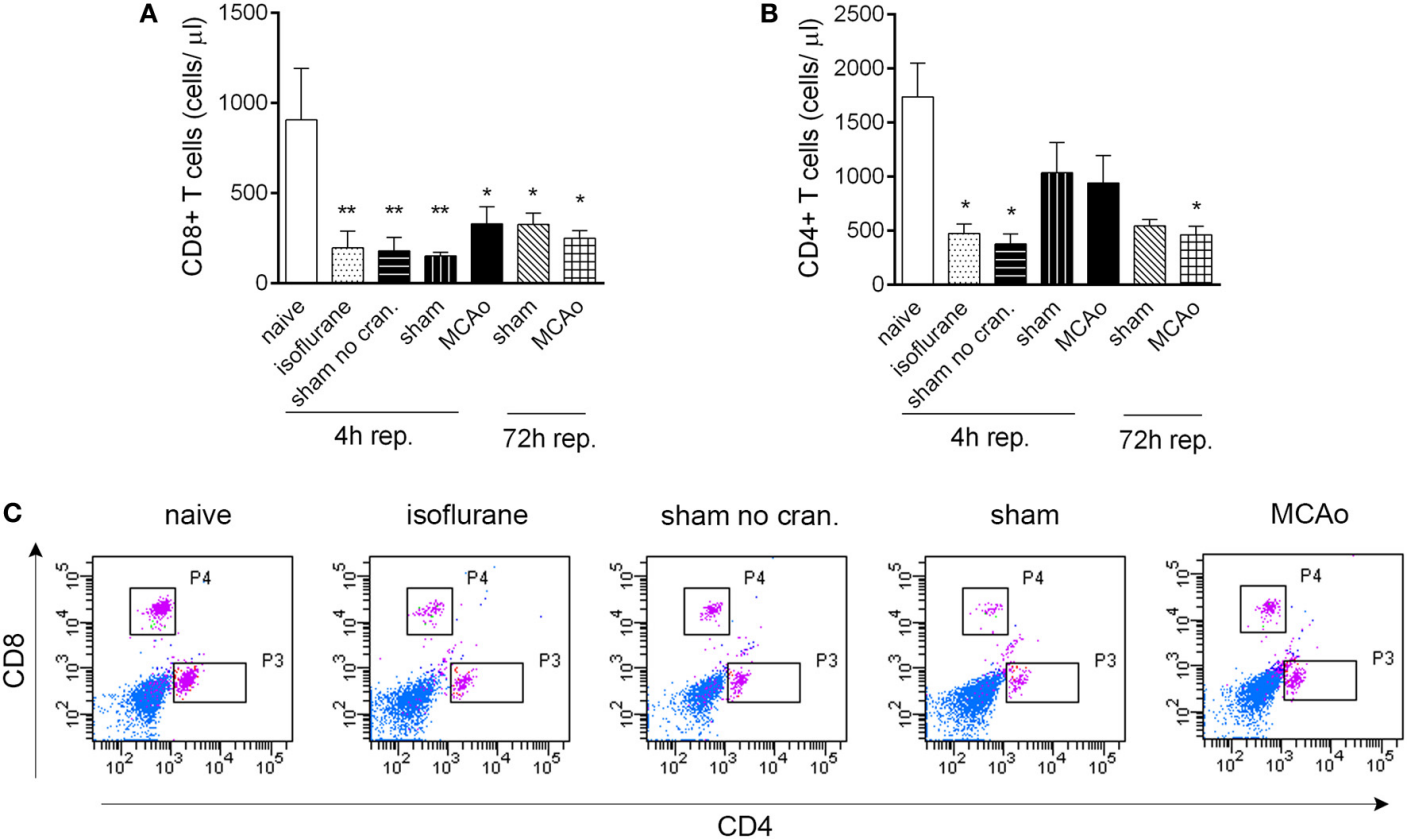
**Isoflurane anaesthesia results in reduced circulating T cell numbers**. CD8+ T cells **(A)** and CD4+ T cells **(B)** are reduced in number in cardiac blood samples within 4 h following isoflurane anaesthesia. **(C)** Representative dot blots showing CD8+ and CD4+ T cells in cardiac blood samples 4 h after reperfusion, control surgeries, or anaesthesia. Purple color indicates cells gated on CD3. Sham no cran.—sham surgery without craniotomy. *n* = 4, 4, 4, 5, 6, 3, 3 in naïve, isoflurane, sham no cran., sham, MCAo, sham 72 h, and MCAo 72 h, respectively. ^*^P < 0.05, ^**^P < 0.01 vs. naive. Light blue color indicates negative (unstained) population.

CD19+ MHCII+ B cells decreased profoundly (by 80–90%) in the blood in response to anaesthesia alone, which was apparent in all other groups of mice that underwent surgical manipulation or MCAo, at 4 h reperfusion (Figures 1F,G). Similarly, anaesthesia alone resulted in reduced circulating CD4+ and CD8+ T cell numbers within 4 h (Figures 2A–C). Interestingly, craniotomy and MCAo appeared to raise T cell numbers over levels after anaesthesia alone, which was more apparent in the case of CD4+ T cells (Figures 2B,C). Circulating B cells and CD8+ T cells remained significantly lower in number in surgery control groups and after MCAo compared to naïve mice at 72 h reperfusion (Figures 1F,G, 2A,C).

### Granulocyte release from the bone marrow increases proportionally to the level of surgical stress and brain injury

**Figure 3:**
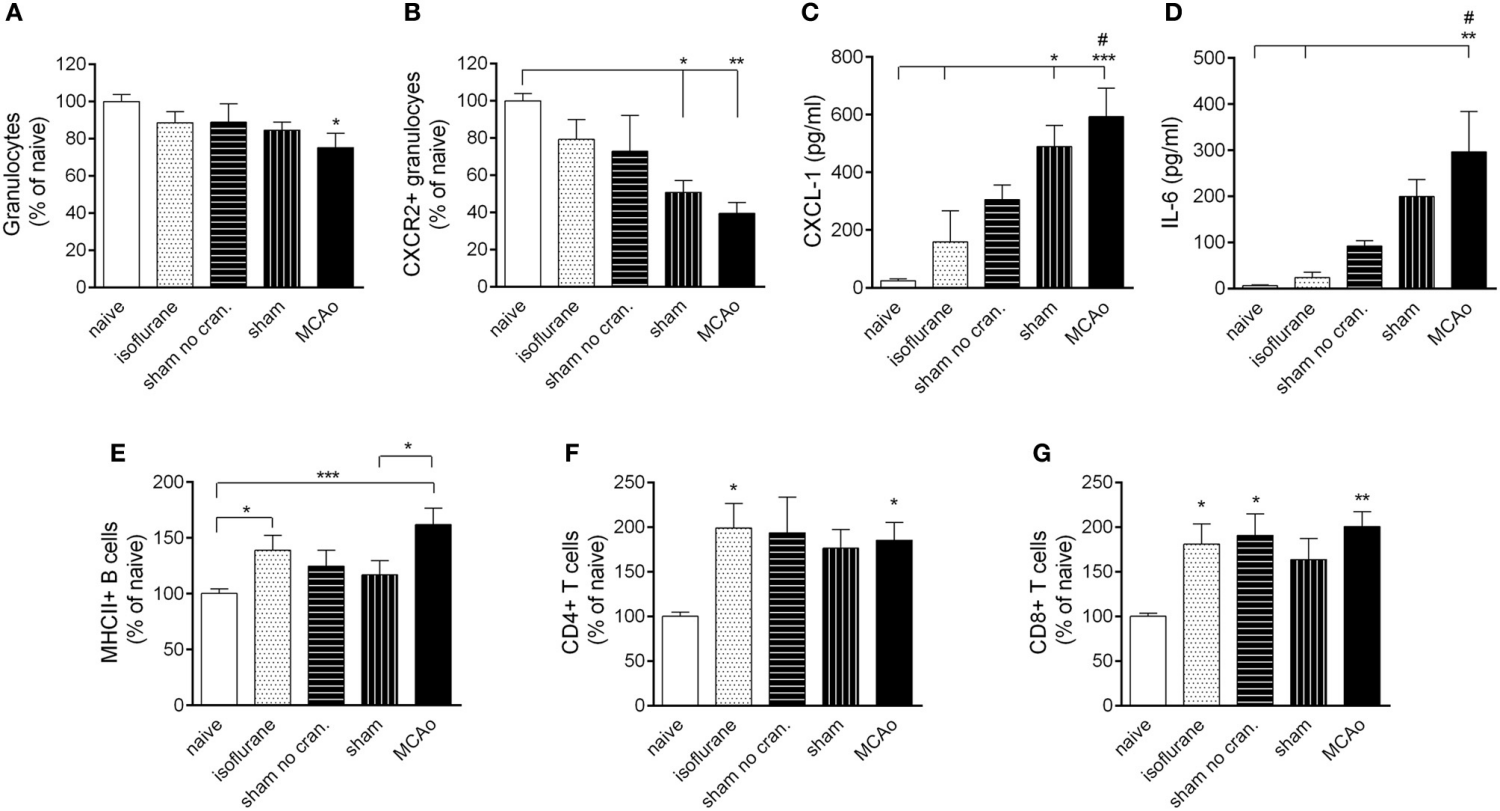
**Surgical intervention and anaesthesia result in altered leukocyte responses in the bone marrow. (A)** Bone marrow granulocytes (Ly6G+, CD11b+, SSC^high^ cells) are reduced within 4 h after MCAo. **(B)** Both MCAo and craniotomy result in reduced CXCR2+ granulocytes in the bone marrow at 4 h reperfusion. KC (CXCL1, **C**) and IL-6 **(D)** levels increase in the circulation within 4 h proportionally to the amount of surgical stress. Anaesthesia results in increased MHCII+ B cells **(E)** CD4+ T cells **(F)** and CD8+ T cells **(G)** in the bone marrow within 4 h. Sham no cran.—sham surgery without craniotomy. *n* = 7, 5, 6, 7, 7–8 in naïve, isoflurane, sham no cran., sham, and MCAo, respectively. ^*^P < 0.05, ^**^P < 0.01, ^***^P < 0.001 vs. naïve, unless indicated otherwise on the graphs; ^#^*P* < 0.05 vs. isoflurane.

MCAo resulted in mobilization of granulocytes from the bone marrow at 4 h reperfusion (Figure 3A). Within the granulocyte population, CXCR2+ granulocytes (Ly6G+ CD11b+ SSC^high^ cells) showed a decrease in the bone marrow proportional to the level of surgical stress, reaching a significant 50% reduction after craniotomy and an over 60% reduction in response to MCAo (Figure 3B). Correspondingly, KC (CXCL-1) and IL-6 concentrations in the circulation increased by 20–40-fold after sham surgery and MCAo (Figures 3C,D). Circulating KC levels showed a significant (*P* < 0.01) negative correlation with numbers of CXCR2+ granulocytes in the bone marrow at 4 h reperfusion (not shown). Anaesthesia alone appeared to increase B cells and T cells in the bone marrow (Figures 3E–G) similarly to our earlier findings (Denes et al., 2011).

### Bone marrow cells do not exhibit a suppressed response after cerebral ischemia

**Figure 4:**
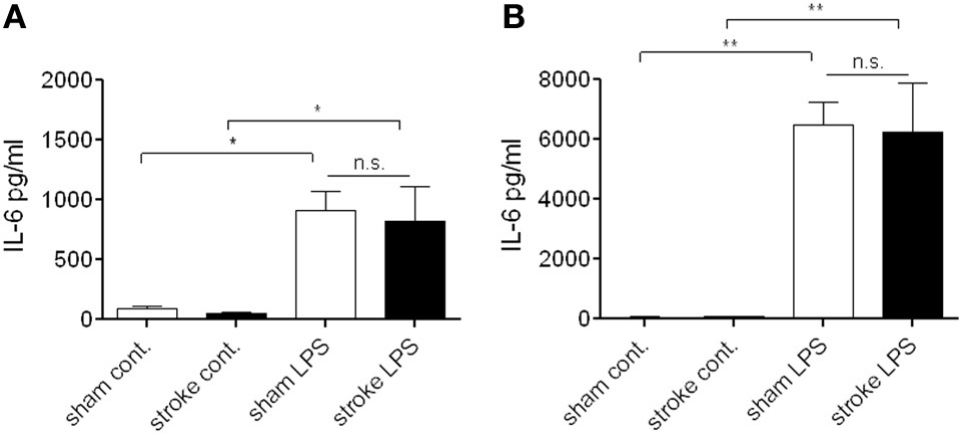
**Bone marrow cells do not exhibit a suppressed response to endotoxin after distal tMCAo**. Bone marrow cells were isolated 72 h after MCAo and stimulated *in vitro* with bacterial lipopolysaccharide (LPS) to investigate cytokine production. Cell lysates **(A)** and supernatants **(B)** were measured for IL-6 production and release. n.s., not significant. *n* = 3. ^*^P < 0.05, ^**^P < 0.01 vs. control.

At 72 h reperfusion when systemic immunosuppression is apparent after cerebral ischemia (Prass et al., 2003), bone marrow cells showed no difference in LPS-induced IL-6 production *in vitro* as assessed in cell lysates (Figure 4A) and cell culture supernatants (Figure 4B), indicating that at this time point cells residing in the bone marrow maintain their ability to respond to endotoxin.

### Surgical intervention and cerebral ischemia alter splenic granulocyte responses

**Figure 5:**
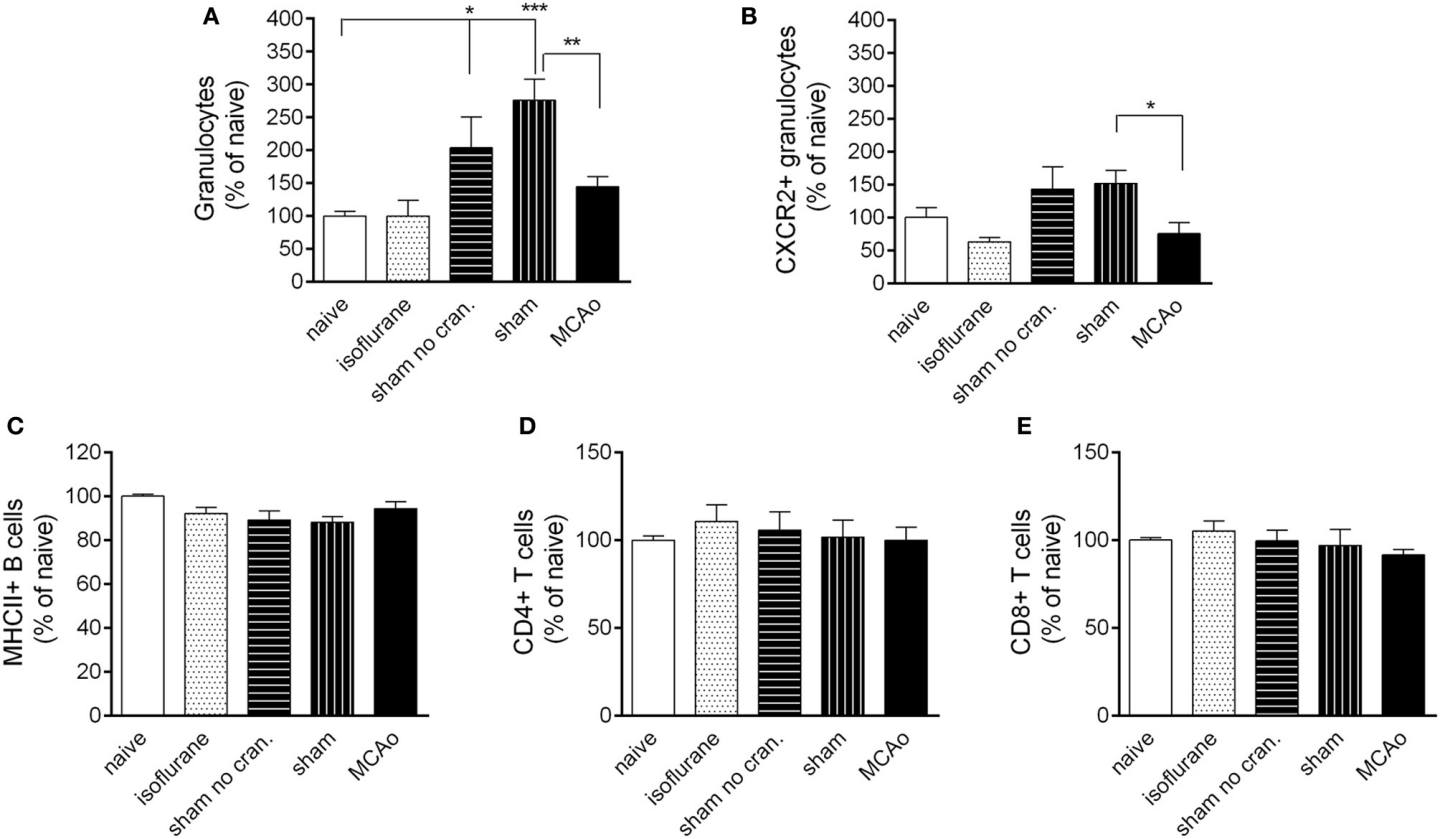
**Leukocyte responses in the spleen**. Changes in total granulocytes (Ly6G+, CD11b+, SSC^high^ cells, **A**) and CXCR2+ granulocytes **(B)** in the spleen 4 h following reperfusion or control surgical interventions. No changes are observed in splenic MHCII+ B cells **(C)** CD4+ T cells **(D)** and CD8+ T cells **(E)** at 4 h reperfusion. Sham no cran.—sham surgery without craniotomy. *n* = 6, 6, 6, 5, 6 in naïve, isoflurane, sham no cran., sham, and MCAo, respectively. ^*^P < 0.05 and ^***^P < 0.001 vs. naïve; ^**^P < 0.01 **(A)** and ^*^P < 0.05 **(B)** sham vs. MCAo.

Surgical manipulation of extracranial tissues with or without craniotomy resulted in an early recruitment of granulocytes in the spleen (Figure 5A). This effect was not apparent in the CXCR2+ granulocyte population (Figure 5B). MCAo significantly reduced splenic total granulocyte- and CXCR2+ granulocyte numbers (Figures 5A,B) compared to sham surgery at 4 h reperfusion. No changes were observed in B cell or T cell populations in the spleen in any of the surgical groups (Figures 5C–E).

### CXCR2 blockade prevents granulocyte mobilization from the bone marrow but does not alter ischemic brain injury

**Figure 6:**
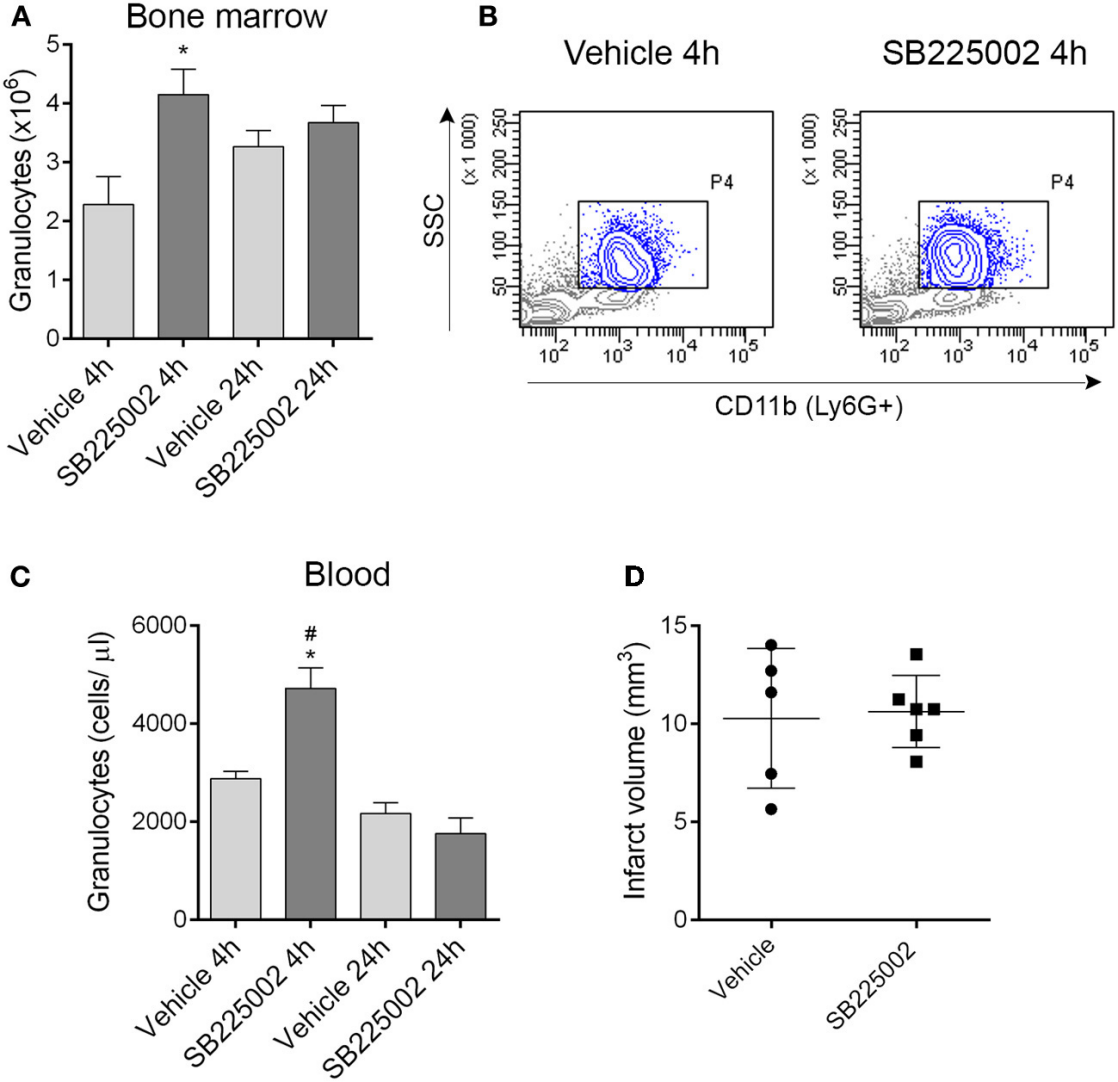
**Blockade of CXCR2-mediated granulocyte release from the bone marrow does not alter infarct size after experimental stroke. (A)** Treatment with the selective CXCR2 antagonist SB225002 prevents granulocyte release from the bone marrow after MCAo compared to vehicle at 4 h reperfusion. Total granulocyte numbers recovered from 1 femur and 1 tibia are shown. **(B)** Representative density plots showing CD11b+ SSC^high^ granulocytes (gated also on Ly6G+, blue) in the bone marrow at 4 h reperfusion. **(C)** SB225002 increases granulocyte numbers in the cardiac blood at 4 h reperfusion after MCAo. **(D)** Infarct size is not altered in response to SB225002 treatment at 24 h reperfusion, after MCAo. **(A–C)** *n* = 3, 3, 5, 5 in vehicle 4 h, SB225002 4 h, vehicle 24 h, and SB225002 24 h samples, respectively, **(D)** *n* = 5–6. ^*^P < 0.05 vs. vehicle 4 h, ^#^*P* < 0.05 vs. 24 h time points.

The selective CXCR2 inhibitor SB225002 (White et al., 1998) prevented granulocyte release from the bone marrow as confirmed by a significant reduction of granulocytes in the bone marrow in vehicle-treated mice 4 h after MCAo compared to mice treated with SB225002 (Figures 6A,B). Similarly to our earlier experiments using the intraluminal filament method to induce cerebral ischemia (Denes et al., 2011) bone marrow granulocyte numbers were stabilized by 24 h post MCAo (Figure 6A). To our surprise, CXCR2 blockade although reducing granulocyte release from the bone marrow, resulted in an increase in circulating granulocytes at 4 h reperfusion compared to vehicle (Figure 6C). We investigated whether CXCR2 blockade resulted in any changes in infarct size, but no difference between SB225002 treatment and vehicle was observed (Figure 6D), indicating that CXCR2-mediated signals do not contribute substantially to brain injury in the current experimental model of cerebral ischemia.

## Discussion

Here we present evidence that even mild surgical manipulation results in marked systemic granulocyte mobilization responses. Granulocyte mobilization is proportional to the level of surgical stress and is further augmented by brain injury. Granulocyte responses to cranial injury or cerebral ischemia involve primarily the mobilization of CXCR2-positive granulocytes, which is not seen after manipulation of extracranial tissues. In addition, we show that anaesthesia profoundly alters leukocyte responses, an effect that lasts for several hours or even days. Collectively, these data highlight the role of anaesthesia and surgical intervention in systemic inflammatory responses, which could have an impact on experimental models of cerebral ischemia, but could also serve important information for the management of patients subjected to various surgeries in the clinic.

In our previous study we revealed changes in the bone marrow in response to transient, focal cerebral ischemia induced by an intraluminal filament that is advanced through the internal carotid artery to occlude the MCA (Denes et al., 2011). Although we demonstrated changes induced by brain injury itself, we also showed that anaesthesia and surgical intervention could impact on bone marrow leukocyte responses (Denes et al., 2011). Since the filament MCAo model involves surgical manipulation around the neck including exposure of the salivary glands, the vagus nerve and other tissues around the trachea, in the present study we performed a systematic analysis of bone marrow, spleen, and blood responses, using a distal MCAo model, to reveal stroke-specific changes in leukocyte mobilization as well as the impact of surgical intervention and anaesthesia. Since the present model includes craniotomy, a potential confounder that disturbs the internal milieu of the brain, we also included control surgeries during which only extracranial tissues were manipulated. Extracranial manipulation consisting of an incision on the skin and temporary dislocation of small pericranial muscles was sufficient to generate a systemic inflammatory response evidenced by granulocyte mobilization and a 10-fold increase in circulating KC levels. Since these changes did not involve major alterations in CXCR2+ granulocytes, identification of the mechanisms involved (contribution of bone marrow-derived cells, signals that initiate and maintain systemic granulocyte responses, etc.) warrant further investigation. It is likely that activation of the HPA axis and the autonomic nervous system (Elenkov et al., 2000) could contribute to surgery-induced peripheral changes, however the exact signals mediating leukocyte responses need to be identified. In fact, we confirmed here our earlier findings showing an increased mobilization of CXCR2-positive granulocytes in response to brain injury, compared with other surgical manipulations (Denes et al., 2011).

We found that isoflurane anaesthesia alone resulted in a profound reduction in circulating lymphocytes within hours. Some effects of isoflurane on leukocyte activation have been reported earlier (Yuki et al., 2012; Carbo et al., 2013), and a decrease in Th1/Th2 ratio in the blood has been observed in patients undergoing craniotomy in response to isoflurane (Inada et al., 2004). However, the present study demonstrates a rapid and sustained reduction in circulating T cell numbers induced by isoflurane anaesthesia, which could have important implications clinically and also in models of experimental stroke. The contribution of T cells to the ischemic brain injury is well documented (Iadecola and Anrather, 2011). Since volatile anaesthetics can exert neuroprotective properties (Kawaguchi et al., 2005), it is possible that at least in part, these actions could be mediated via blunted T cell responses.

The spleen is profoundly affected by brain injury (Offner et al., 2006b), leading to loss of B cells and development of immunosuppression, however, much less is known about how cerebral ischemia contributes to responses of myeloid cells in the spleen. In fact, a population of splenic monocytes are released rapidly upon activation and contributes to ischemic processes in the heart (Swirski et al., 2009; Leuschner et al., 2012). We found that surgical manipulation results in increased granulocyte recruitment to the spleen whilst cerebral ischemia reduced surgery-induced increases in splenic granulocytes. Although the role and mechanisms of surgery-induced splenic granulocytosis requires further investigations, this scenario indicates that the effect of surgical manipulation on leukocyte responses in peripheral organs is likely to be a confounder in current experimental stroke models.

CXCR2 is the best characterized cell surface receptor on granulocytes involved in cell mobilization to stimuli mediated by CXCL1 (KC) and CXCL2/3 (MIP-2) (Matzer et al., 2004; Eash et al., 2010; Veenstra and Ransohoff, 2012). Although brain injury-induced granulocyte mobilization from the bone marrow was prevented by CXCR2 blockade, circulating granulocytes increased in response to SB225002 treatment. CXCR2 inhibition was found effective to reduce IL-8- or KC-induced granulocyte mobilization in the blood and block granulocyte egress from the bone marrow (White et al., 1998; Martin et al., 2003; Eash et al., 2010). We show here that very early granulocyte mobilization responses in response to brain injury are not dependent on CXCR2, and might not (or only in part) require the contribution of the bone marrow in the current experimental model. This could explain the lack of an effect by CXCR2 blockade on the size of ischemic brain injury, which has also been confirmed by another study (Brait et al., 2011). Brain injury in different experimental models might not be influenced by peripheral leukocyte actions to the same extent. For example, blockade of granulocyte responses or hematopoietic MyD88-dependent actions is not associated with reduced central inflammation or brain damage after cold-induced cortical injury (Koedel et al., 2007). In contrast, neutrophils contribute to brain injury when experimental stroke is preceded by systemic inflammation (McColl et al., 2007). Therefore, the source of neutrophils mobilized after brain injury and their contribution to the population that is recruited into the brain need to be investigated in further studies. Stroke in patients results in increased circulating granulocyte numbers and loss of T cells (Vogelgesang et al., 2008), which corresponds to the findings presented in our study. Surgical interventions and anaesthesia also influence leukocyte responses in patients (Inada et al., 2004). However, similarly to most experimental stroke models, the MCAo model is inherently confounded by effects of anaesthesia and surgical intervention, and we show that surgical interventions alone are capable of inducing profound changes in leukocyte responses. These changes might contribute to and/or alter brain injury in experimental models. Since circulating white blood cell- and blood cytokine data from stroke patients and from those undergoing anaesthesia or surgical interventions are widely available, blood cell responses and mechanisms of brain injury in experimental models should be investigated in a translational context.

In conclusion, our data indicate that anaesthesia- and surgery-induced leukocyte responses interact with those induced by experimental stroke, which needs to be accounted for in experimental models. Revealing the mechanisms of rapid, surgery- or brain injury-induced granulocyte responses could be fundamental for the development of new therapeutic regimen in a wide variety of diseases.

### Conflict of interest statement

The Reviewer Dr. Jaleel A. Miyan declares that, despite being affiliated to the same institution as the authors, the review process was handled objectively. The other authors declare that the research was conducted in the absence of any commercial or financial relationships that could be construed as a potential conflict of interest.
